# Galactosialidosis: review and analysis of *CTSA* gene mutations

**DOI:** 10.1186/1750-1172-8-114

**Published:** 2013-08-02

**Authors:** Anna Caciotti, Serena Catarzi, Rodolfo Tonin, Licia Lugli, Carmen Rodriguez Perez, Helen Michelakakis, Irene Mavridou, Maria Alice Donati, Renzo Guerrini, Alessandra d’Azzo, Amelia Morrone

**Affiliations:** 1 Laboratory for Molecular and Cell Biology, Paediatric Neurology Unit and Laboratories, Meyer Children’s Hospital, Viale Pieraccini n. 24, Florence 50139, Italy; 2 Department of Neurosciences, Psychology, Pharmacology and Child Health, University of Florence, Viale Pieraccini 24, Florence, Italy; 3 U.O. Neonatology, Azienda Ospedaliera Policlinico di Modena, Via del Pozzo 71, Modena, Italy; 4 U.O. Neonatology, A.O. Spedali Civili, Presidio Umberto I, Brescia, Italy; 5 Department of Enzymology and Cellular Function Institute of Child Health, Aghia Sophia Children’s Hospital, GR-11527 Athens, Greece; 6 Metabolic and Muscular Unit, Meyer Childrens’ Hospital, Viale Pieraccini 24, Florence, Italy; 7 Department of Genetics, St Jude Children’s Research Hospital, 262 Danny Thomas Pl, Memphis, TN 38105, USA

## Abstract

**Background:**

Mutations in the *CTSA* gene, that encodes the protective protein/cathepsin A or PPCA, lead to the secondary deficiency of β-galactosidase (GLB1) and neuraminidase 1 (NEU1), causing the lysosomal storage disorder galactosialidosis (GS). Few clinical cases of GS have been reported in the literature, the majority of them belonging to the juvenile/adult group of patients.

**Methods:**

The correct nomenclature of mutations for this gene is discussed through the analysis of the three PPCA/CTSA isoforms available in the GenBank database. Phenotype-genotype correlation has been assessed by computational analysis and review of previously reported single amino acid substitutions.

**Results:**

We report the clinical and mutational analyses of four cases with the rare infantile form of GS. We identified three novel nucleotide changes, two of them resulting in the missense mutations, c.347A>G (p.His116Arg), c.775T>C (p.Cys259Arg), and the third, c.1216C>T, resulting in the p.Gln406* stop codon, a type of mutation identified for the first time in GS. An Italian founder effect of the c.114delG mutation can be suggested according to the origin of the only three patients carrying this mutation reported here and in the literature.

**Conclusions:**

In early reports mutations nomenclature was selected according to all *CTSA* isoforms (three different isoforms), thus generating a lot of confusion. In order to assist physicians in the interpretation of detected mutations, we mark the correct nomenclature for CTSA mutations. The complexity of pathology caused by the multifunctions of CTSA, and the very low numbers of mutations (only 23 overall) in relation to the length of the *CTSA* gene are discussed.

In addition, the *in silico* functional predictions of all reported missense mutations allowed us to closely predict the early infantile, late infantile and juvenile phenotypes, also disclosing different degrees of severity in the juvenile phenotype.

## Background

The protective protein/cathepsin A (PPCA or CTSA) is a multifunctional lysosomal enzyme with distinct protective and catalytic function [1].

The mature form of PPCA/CTSA, consisting of a 32/20 kDa disulfide-linked two chain product, is found in a high molecular weight, lysosomal multienzyme complex (LMC) together with two other glycosidases, β-galactosidase (GLB1) and N-acetyl alpha neuraminidase 1 (NEU1) [2-4]. A supposed role of N-acetylgalactosamine-sulfate sulfatase (GALNS) in such complex [5] still lacks interpretation.

Association with PPCA/CTSA in an early biosynthetic compartment ensures the correct lysosomal transport, activation and stability of GLB1 and NEU1 [6]. This defines the enzyme protective function. On the other hand, studies of the physiological role of PPCA/CTSA as a serine carboxypeptidase/deamidase/esterase demonstrated a role of the enzyme in the inactivation of selected neuropeptides, like substance P, oxytocin and endothelin I [7]. PPCA/CTSA is also responsible for the proteolytic inactivation of Lysosome-associated membrane protein (LAMP)2a, a lysosomal integral membrane protein involved in chaperone mediated autophagy, thus regulating this lysosomal pathway of protein degradation [8].

Mutations in the *CTSA* gene are the cause of the lysosomal storage disease galactosialidosis (GS). Loss of function of PPCA/CTSA results in the secondary combined deficiency of GLB1 and NEU1, which is the biochemical hallmark of the disease. Patients with GS present with a broad spectrum of clinical manifestations, but are usually classified as early infantile, late infantile or juvenile/adult type based on the age of onset and severity of their symptoms.

The early infantile phenotype, the most severe form of the disease, usually presents with hydrops fetalis, cherry red spots, visceromegaly, psychomotor delay, coarse facies, skeletal dysplasia, and early death. Late infantile forms are characterized by corneal clouding, cardiac involvement, visceromegaly and, rarely, psychomotor retardation. Most patients with the milder juvenile/adult form, exhibited myoclonus, ataxia, neurological deterioration, angiokeratoma, and absence of visceromegaly [9,10].

It is still unclear whether the catalytic function of PPCA/CTSA contributes to particular clinical signs of GS. In this respect, impaired LAMP2a degradation due to PPCA/CTSA deficiency may be linked to the low weight of affected individuals [8] and the lack of inactivation of specific bioactive peptides may play a role in the regulation of the blood pressure [7].

In addition, it has been previously suggested that PPCA/CTSA plays a role in elastic fibers assembly, through its association with the enzymatically inactive, spliced variant of β- galactosidase, known as the elastin binding protein (EBP). Because EBP acts as an intracellular chaperone for tropoelastin, facilitating the trafficking and deposition of elastic fibers [11], lack of PPCA/CTSA in GS can be accompanied by alterations in elastogenesis, affecting the cardiovascular and respiratory systems [7,12,13]. This is especially true for GS patients with a longer survival, as they need periodic assessment of their pulmonary function and emphysema, linked to a defect in elastic fiber assembly [10].

Because PPCA/CTSA is present in the LMC, mutations altering the folding of one protein in the complex can influence the other components as well [14,15]. Multiple sequence alignments may predict functional sites or domains that may favor intra- or inter-molecular interactions within the LMC. Analogously, structural web applications may predict an amino acid substitution as disease-causing or neutral in humans, and it could suggest the molecular causes of a disease. For instance, gain of helical propensity or loss of a phosphorylation site or disorder to order transitions caused by Molecular Recognition Features (Morf), specific regions of proteins that exhibit molecular binding functions [16].

A total of 23 *CTSA* gene mutations have been reported (HGMD professional https://portal.biobase-international.com/cgi-bin/portal/login.cgi). These include deletions, missense and splicing mutations, but no nonsense mutations. Alternatively spliced transcripts of the *CTSA* gene have been described, and at least three *CTSA* mRNA sequences have been deposited in the GenBank database (RefSeq, http://www.ncbi.nlm.nih.gov/gene/5476). In early reports mutations nomenclature was selected according to all *CTSA* isoforms, which generated confusion in the way *CTSA* mutations were reported. Some mutations were numbered based on a PPCA sequence that was either 46 (missing all the amino acids of the signal peptide) [17-20] or 18 [13,21] amino acids shorter than that reported in the HGMD professional database.

In order to assist physicians in the interpretation of detected mutations, we review and discuss previously reported CTSA transcripts, underlying erroneous or current nomenclature. We also present the molecular and clinical assessment of four new observations of the rare infantile form of GS. Computational analyses to predict a role of the new and/or previously reported missense mutations are discussed in order to address the clinical and molecular implications of the CTSA defects in GS patients.

## Methods

### Cell culture

Skin fibroblasts from patient 3, and normal controls were cultured in Ham’s F-10 medium supplemented with 10% fetal bovine serum and antibiotics. Cell lines from the remaining patients we report are no longer available.

### Enzyme assays

The Micro BCA protein Assay kit (Pierce Rockford, USA) was used to set up the starting proteins used in each enzyme assay. GLB1, NEU1, PPCA and GALNS activities were measured in cell lysates of fibroblasts and/or leukocytes using commercially available fluorogenic substrates (Moscerdam substrates, Netherlands) and according to manufacturer’s instructions (http://www.moscerdam.com). For NEU1 assays: cells lysates, BCA measurements and assays were performed in fresh samples, lysed by pipetting.

### PCR amplification of genomic DNA and informed consents

Genomic DNA was extracted from the patient’s fibroblasts and/or lymphocytes using standard methods. The genomic fragments covering all 15 exons and the exon/intron boundaries of the *CTSA* gene were amplified by a set of primers located in flanking intronic sequences. PCR amplifications were performed under the previously reported conditions [13]. Informed consents for genetic tests were obtained for all analysed patients included in the study.

### DNA sequencing

PCR fragments were separated on a 2% agarose gel and the bands were visualized using a UV transilluminator. DNA products were purified by Nucleospin Extract kit (Macherey-Nagel, Düren, Germany), following the manufacturer’s protocol. The double-stranded purified products were used for direct sequencing with the same PCR amplification primers. Sequencing reactions were performed using the ABI PRISM 3130 Genetic Analyzer (Applied Biosystems, Foster City, U.S.A.) as recommended by the manufacturer.

### Screening of new mutations and in silico analyses

The 1000 Genomes project database (http://www.1000genomes.org/) including all human genetic variations from the dbSNP short genetic variations database (http://www.ncbi.nlm.nih.gov/omim) did not report the frequency of the newly presented point mutations. In order to analyse the actual frequency of such variants in the Italian population, the *CTSA* gene of 60 normal control DNA samples was analysed by sequencing analysis. The PCR fragments were amplified by the genomic primers reported earlier [13]. In addition, the single amino acid substitutions were also analysed by SIFT (http://sift.jcvi.org/www/SIFT_aligned_seqs_submit.html) and PolyPhen (http://genetics.bwh.harvard.edu/pph/) multiple sequence alignments of CTSA related proteins.

The functional effect of novel missense mutations on resulting CTSA enzymes was predicted by MutPRed (http://mutpred.mutdb.org/) web site.

## Results

### Clinical and biochemical data of the four GS patients here reported

**Figure 1:**
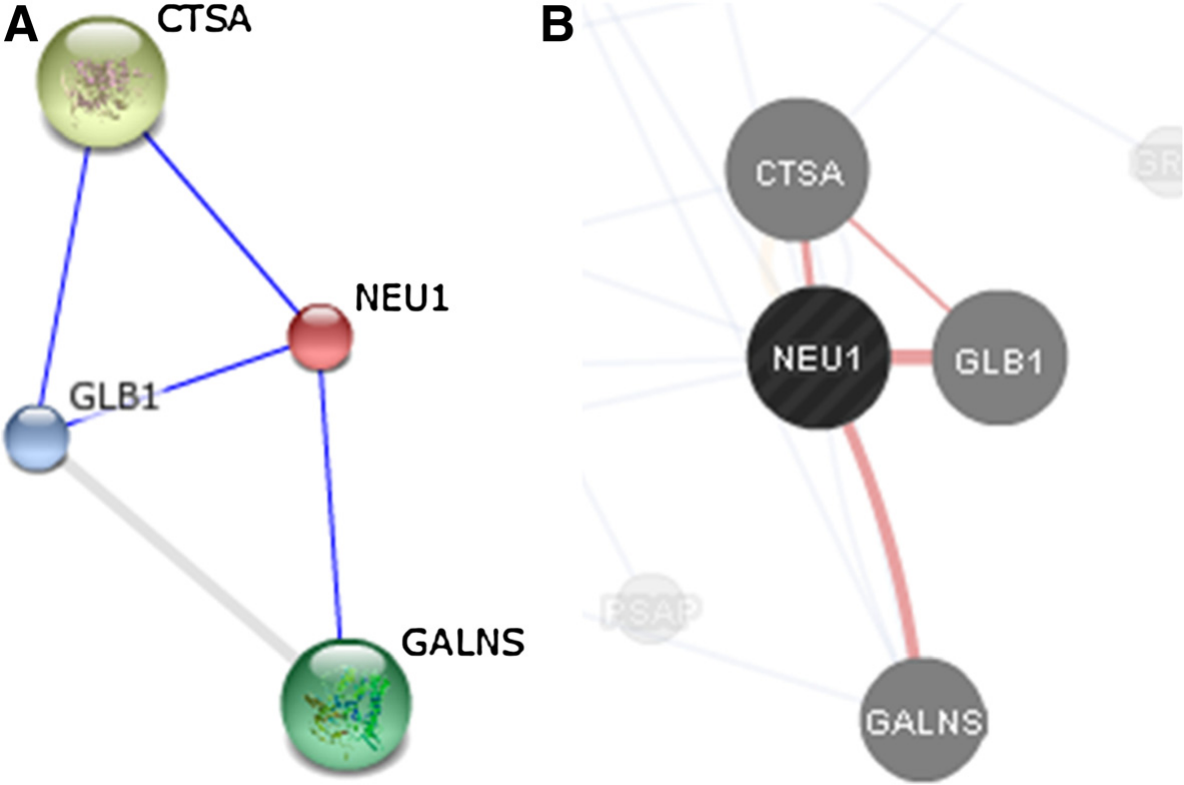
**Action view of the physiological interactions between GALNS, GLB1, NEU1 and PPCA/CTSA in human cells. A**. String prediction; **B**. Gene Mania prediction.

The clinical data and mutation analysis of the new GS patients reported here are summarised in Table 1. All patients showed vacuolated lymphocytes in peripheral blood smears. A marked reduction of GLB1 and absence of NEU1 activity confirmed the diagnosis of GS on fibroblasts and/or lymphocytes (Table 1). Biochemical data were performed in different accredited diagnostic laboratories (thus control values are different), but NEU1 activity was nearly absent in all patients while patients’ GLB1 activity ranged from 2 to 19% of normal values. CTSA activity, measured in Pt3 fibroblasts, was also completely absent (normal value 191- 482 nmol/mg/min). In contrast, GALNS assays performed in both lymphocytes and fibroblasts of Pt3 was within the normal range. Indeed, functional protein association networks as Gene Mania (http://www.genemania.org/) and String (http://string-db.org/) showed that GALNS interacts at least with NEU1 but not directly with PPCA/CTSA inside the LMC (Figure 1).

**Table 1:** Clinical findings of galactosialidosis patients

**Patient**	**1**	**2**	**3**	**4**
**Ethnic Origin**	Caucasian	Caucasian	Caucasian	Caucasian
**Sex**	F	M	M	M
**Clinical Phenotype**	EI	EI	EI	EI
**Age at Onset**	perinatal	perinatal	perinatal	perinatal
**Fetal hydrops**	+	-	+	-
**Edema**	+	-	+	+ (1 month of age)
**Presentation**	fetal hydrops, developmental delay	failure to thrive since birth	fetal hydrops	respiratory distress, inguinal hernia, telangectasias, equinovarus feet, arthrogryposis, gingival hypertrophy
**Age at diagnosis**	4 m	17 m	1 m	post-mortem
**Psychomotor delay**	+	+	+	+
**Hypotonia**	+	-	+	+
**Course facies**	+	+	+	+
**Eye**	lens clouding	-	-	hypopigmentated fundus
**Hepatosplenomegaly**	+	+	+	-
**Cardiac involvement**	+	-	+	+
**Skeletal involvement**	-	+	-	+
**Renal involvement**	-	no	no	no
**Seizures**	no	no	no	no
**Muscle involvement**	no	no	no	no
**Brain MRI**	-	-	enlarged ventricles, hyperintense white matter	striato thalamic vasculopathy, widened periencephalic spaces
**GLB1 activity (nmol/mg/h)**	27 (NV 391-2397) on fibroblasts	61 (NV 391-2397) on fibroblasts	143 (NV 400-1100) on fibroblasts; 12 (NV 90-250) on leukocytes	44,1 (NV 291-525) on fibroblasts
**NEU1 activity (nmol/mg/h)**	0,1 (NV 5,1-48) on fibroblasts	0,76 (NV 5,1-48) on fibroblasts	0 on fibroblasts (NV 17- 68) and leukocytes (NV 0,37- 3)	2,4 (NV 66-197) on fibroblasts
**Other**	-	-	dysphagia telangectasias, inguinal hernia, recurrent pleural effusion and ascites, clubfeet	failure to thrive, frequent vomiting, ascites, hypertelorism, thrombocytopenia
**Alive (age)**	deceased	deceased	deceased at 3 m	deceased at 4 m, 22d
***CTSA *molecular analysis**	c.448C > A (p.Val150Met)/c.1216C > T (p.Gln406*)	c.775 T > C (p.Cys259Arg)/c.775 T > C (p.Cys259Arg)	c.114delG/c.347A > G (p.His116Arg)	c.114delG/c.114delG

Legend: *F* female, *M* male, *EI* early infantile, + presence of the symptom, - not available data; *no* normal, *m* months, *d* days, *NV* normal values. New mutations are evidenced.

Common symptoms in all four patients included facial dysmorphisms, hypotonia, developmental delay, failure to thrive, liver-spleen-megaly and ascites (Table 1). Respiratory distress and cardiac involvement, when investigated, were also observed. Cardiomyopathy was detected in Pt1. Pt3 was intubated for respiratory failure at birth. He also exhibited reduced cardiac contractility and cardiac dilatation with stiff and dilated inferior vena cava at the 2nd day of life. Pt4 presented with hypertrophic interventricular septum, which evolved into dilated cardiomyopathy. Skeletal involvement was also noticed in this patient since he was one week old. He exhibited a rarefaction of femoral, tibial and heels metaphysis together with horizontalysed acetabula. Brain magnetic resonance imaging performed in Pt3 and 4, identified alterations in brain structure and blood flow (Table 1).

### *In silico* evaluation of CTSA isoforms and mutation nomenclature

**Figure 2:**
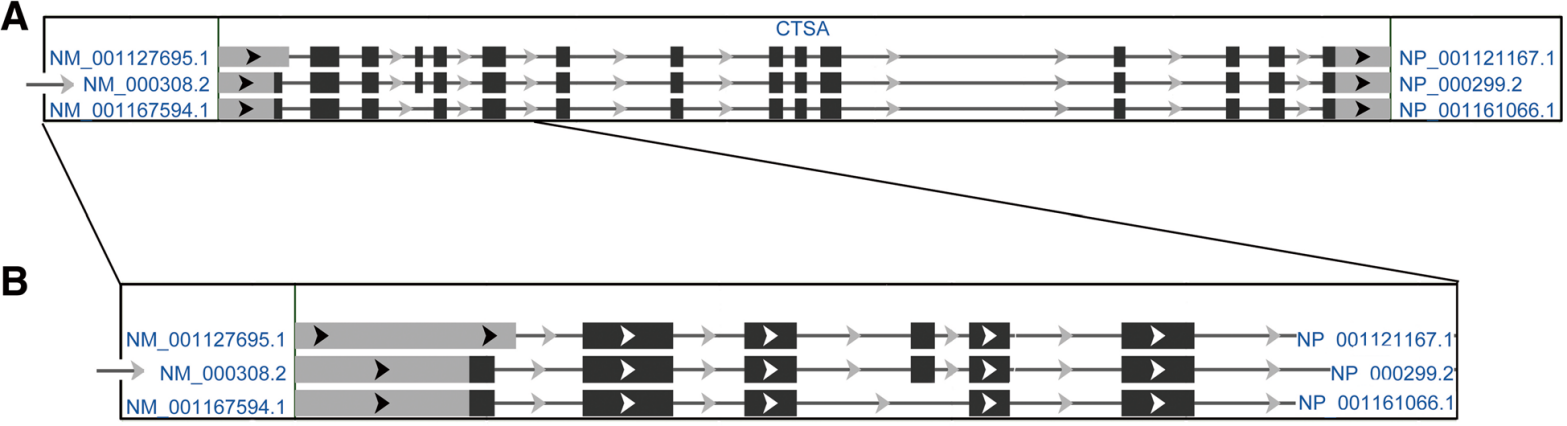
**Schematic representation of the *CTSA *gene transcript variants (from**http://www.ncbi.nlm.nih.gov/). The reference transcript for the correct mutations nomenclature is indicated by an arrow. **A**. Transcript Variant 1 encoding the longer isoform (a) of 498 amino acids (NM_000308.2) is the gene transcript selected by HGMD for mutations nomenclature. Transcript Variant 2 has an alternate splice site in the 5′ region, which results in a downstream AUG start codon, as compared to variant 1 (NM_001127695.1). The resulting isoform (b), gives rise to 480 amino acids. Transcript Variant 3 lacks an alternate in-frame exon in the 5′ coding region, compared to variant 1 (NM_001167594.1). **B**. The main differences between the three transcripts are outlined.

In order to avoid confusion in mutation nomenclature, we critically examine reported CTSA variants (RefSeq, http://www.ncbi.nlm.nih.gov/gene/5476). Transcript variants and their differences are outlined in Figure 2.

Since Variant 1 has been chosen as the reference sequence for the mutation nomenclature in HGMD professional (https://portal.biobase-international.com/cgi-bin/portal/login.cgi), reported mutations have to be listed according to NM_000308.2 CTSA variant (variant 1, isoform a), as in the current report.

### Sequencing analysis of the *CTSA* gene

The results of mutation analyses of the GS patients reported here are summarised in Table 1.

Mutation analysis was performed by direct sequencing of all 15 exons, including the signal peptide sequence and the intron/exon boundaries of the patients’ *CTSA* gene. Three new point mutations were identified: c.1216C>T (p.Gln406*) c.775T>C (p.Cys259Arg) and c.347A>G (p.His116Arg). Computational analyses of the known point mutations (reported in HGMD professional database), and of the new point mutations identified are reported in Table 2.

**Table 2:** CTSA gene point mutations and significance

**Mutation**	**Ex.**	**Phe.**	**Mut Pred computational analysis**				**PPCA three-dimensional reported outputs (Zhou et al. 1996 [**[22]**]; Rudenko et al. 1998 [**[19]**]; Takiguchi et al 2000 [**[23]**])**	**Philogenetic conservation**		**Ref.**
			**P.**	**Actionable hypotheses**	**Confident hypothesis**	**Very confident hypothesis**		**SIFT score**	**Polyphen score**	
				***(g*** **> 0.5, *p*** **< 0.05)**	**(*g*** **> 0.75, *p*** **< 0.05)**	**(*g*** **> 0.75, *p*** **< 0.01)**				
p.Gln67Arg	2	EI	0.941		Gain of MoRF binding		Drastically alters folding and stability	T (0,07)	D (0,996)	Shimmoto 1993 [21]
p.Ser69Tyr	2	EI	0.942		Loss of disorder		Drastically alters folding and stability	D (0,00)	D (1,000)	Zhou 1996 [22]
p.Val83Arg	2	EI	0.957		Gain of methylation	Gain of disorder	Drastically alters folding and stability	D (0,00)	D (1,000)	Shimmoto 1993 [21]
p.Gly103Ser	3	EI	0.974					D (0,00)	D (1,000)	Groener 2003 [17]
p.Gly103Val	3	EI	0.962					D (0,00)	D (1,000)	Kiss 2008 [18]
p.Ser108Leu	3	EI	0.962			Loss of disorder	Drastically alters folding and stability	D (0,00)	D (1,000)	Shimmoto 1993 [21]
p.His116Arg	3	EI	0.712	Gain of methylation				T (0,45)	D (0,999)	*This work*
p.Val150Met	5	EI	0.947		Loss of catalytic residue		Drastically alters folding and stability	D (0,00)	D (1,00)	Zhou 1996 [22]
p.Leu254Pro	8	EI	0.921		Loss of stability; Gain of catalytic residue		Drastically alters folding and stability	D (0,00)	D (1,000)	Zhou 1996 [22]
p.Cys259Arg	8	EI	0.726	Gain of disorder; Loss of ubiquitination				D (0,00)	D (1,00)	*This work*
Tyr267Asn	8	LI	0.798		Gain of disorder; Loss of stability		Milder effect on protein function and residual mature PPCA	D (0,04)	B (0,189)	Shimmoto 1993 [21]
Tyr413Cys	13	EI	0.933				Drastically alters folding and stability	D (0,00)	D (1,000)	Shimmoto 1993 [21]
p.Met424Tyr	13	EI/LI	0.804				Generation of a glycosylation site and destabilization of dimerisation	D (0,01)	B (0,214)	Zhou 1996 [22]
p.Arg442Trp	14	LI	0.697					D (0,01)	D (1,000)	Kiss 2008 [18]
p.Gly457Ser	14	EI	0.948				Drastically alters folding and stability	D (0,01)	D (1,000)	Zhou 1996 [22]
p.Phe458Val	14	LI	0.867				Milder effect on protein function, destabilization or defective dimerisation of the precursor	T (0,15)	D (0,990)	Zhou 1991 [20]
p.Lys471Glu	14	J/A	0.893		Loss of methylation		Milder effect on protein function, destabilization or defective dimerisation of the precursor	T (0,21)	D (1,000)	Takiguchi 2000 [23]

Legend: *P* Probability of deleterious mutation, *Ex* exon, *T* Tolerated, *D* Deleterious, *B* Benign, *MoRFs* Molecular Recognition Features, *Phe* Clinical phenotype, *EI* early infantilem *LI* late infantile, *J/A* juvenile/adult. Mutations detected in the patients here reported are underlined.

Pt 1 and Pt3 are both heterozygous for a nonsense mutation and a deletion, thus their second allelic mutations, respectively p.Val150Met and p.His116Arg, can be linked to the severe early infantile phenotype (Tables 1 and 2).

### *In silico* CTSA functional/structure predictions of missense mutations

In Table 2 we have reviewed the clinical phenotypes and genotypes of previously reported GS patients and included the functional/structural predictions of newly identified as well as previously described *CTSA* missense mutations (Table 2).

The clinical features of previously reported homozygous patients allowed for a close genotype- phenotype correlation (Table 2). The p.Tyr413Cys mutation was identified in both early infantile and adult patients. However, in the adult patient it was detected in combination with the c.746 + 3A > G change, reported as a mild mutation in homozygotes adult patients [21,24]. Thus, the p.Tyr413Cys mutation underlies the severe phenotype. The p.Tyr267Asn was detected in a “variant” form of an early infantile patient without neurological involvement [21]. The same mutation was later identified in a patient with the late infantile form of the disease [22] and marginal neurological involvement, if any [9,20]. The p.Gln67Arg was first reported in a juvenile patient [21], in combination with a mild mutation. The early infantile phenotype was linked to such mutation by structure analysis [19], which was confirmed by a subsequent clinical report [25].

Prediction tools indicated that the p.Ser69Val, p.Val83Arg and p.Gly103Val, detected in juvenile patients in combination with mild mutations or reported in non-assigned clinical phenotypes, can be linked to severe outcomes. Coarse facies, hepatosplenomegaly, growth retardation and an unusual renal symptomatology were described in a 9-year-old patient who was compound heterozygous for the p.Gly103Val and p.Arg442Trp mutations [18]. Since this patient had normal neurological development, the diagnosis of late-infantile GS can be supposed and probably linked to the p.Arg442Trp mutation. It was reported earlier that the p.Tyr267Asn correlated with a more severe phenotype than that associated with the p.Phe458Val [22] unless both mutations were found in the juvenile/adult form of the disease. This assumption seems to be correct based on previously reported structure prediction [19] (Table 2).

## Discussion

GS is a rare lysosomal storage disease with most of the described patients having the juvenile/adult form [9,10]. The prevalence of GS is unknown; more than 100 observations have been reported but only 23 mutations have been identified so far, including point-mutations and rearrangements, but no nonsense mutations [9,10]. Here we report the first case of GS carrying a *CTSA* gene nucleotide change leading a stop codon mutation. The rarity of such mutation type in the GS patient population is likely attributable to the rare incidence of the early infantile form of the disease.

We also describe two new missense mutations: c.347A>G (p.His116Arg), and c.775T>C (p.Cys259Arg). The p.His116Arg mutation was detected in combination with the c.114delG, a deletion found at the homozygous level also in Pt4 and previously reported in an unrelated patient originating from the same area of central Italy [13]. Since this mutation was not reported in patients with other origin in the literature and due to the small number of previously reported mutations, a founder effect for such one-base deletion can be hypothesized.

As mentioned earlier, three different *CTSA* cDNAs transcripts are deposited in the GenBank database, thus the past literature on *CTSA* mutations referred randomly to the different isoforms, which has been the source of confusion. For example, the p.Val150Met mutation has been first reported as p.Val104Met [22] and then reported as p.Val132Met [23]. We would like to stress the importance of having the *CTSA* mutation nomenclature homologated to the HGMD professional guide lines, which choose *CTSA* Variant 1 as the reference sequence for mutation nomenclature of the CTSA resulting protein.

To deepen the correlation between mutations and their effects on the CTSA protein structure we performed computational analyses using *in silico* tools based on phylogenetic alignments and functional/structural predictions. Multiple alignments of related sequences among organisms and structural web applications help to identify regions or domains that are conserved and may indicate functional constraints.

We found computational analyses to be helpful in improving the determination of the pathognomonic effects of newly identified nucleotide variants and genotype-phenotype correlations. This method has been particularly useful in the case of compound heterozygous mutations reported in mild affected patients or when clinical data are insufficient, i.e. if expression studies and/or structural analysis of compound heterozygous mutations are not available. The reverse process: from a described phenotype to computational analysis showed a good correlation between *in silico* predictions and mutation severity.

We want to emphasize that predictive functional/structural analyses of mutant proteins and phenotypes are closely related, often providing clearcut indications on the specific form of the disease (early infantile, late infantile, juvenile). We found such analyses of use to predict the extent of disease severity related to two mutations p.Tyr267Asn and p.Phe458Val found in juvenile GS patients. In contrast, phylogenetic comparisons in most cases provide indications that are limited to the pathogenic/non-pathogenic effects of mutations without further details. Thus, the combination of multiple computational analyses is an effective strategy.

CTSA binds and regulates GLB1 and NEU1 inside lysosomes [6]. Structure predictions, identifying mutations that alter Molecular Recognition Features (MoRFs) or result in gain or loss of function in CTSA, could accordingly provide information on the three-dimensional structure of the complex proteins. These findings might provide indications on the pathogenetic effects of mutations and on the interactions between the proteins within the LMC.

Known and predicted protein-protein interactions evaluated by String and GeneMania prediction software evidenced that PPCA/CTSA does not directly interact with GALNS thus corroborating the molecular data showing that the PPCA/CTSA deficit does not affect the GALNS activity in Pt3. However our findings can not exclude the involvement of GALNS in the LMC after the binding with NEU1.

The protective function of PPCA dramatically affects both GLB1 and NEU1, leading to a broad range of clinical manifestations, worsening with age [2]. PPCA defects causes both glycosidases to malfunction, indeed early infantile forms of GS share clinical signs observed in the infantile forms of both GM1 gangliosidosis and type II sialidosis [19]. In addition, altered catalytic activity of PPCA could contribute to the variability of symptoms due to the potential esterase/deamidase activity of PPCA in platelets, endothelial cells, heart and kidney [3]. A cardiovascular role of PPCA could also be linked to the altered function of EBP due to structural mutations of PPCA as its binding partner at the plasma membrane [11]. Our prediction tools identified only one CTSA amino acid change (p.Val150Met) that could affect its catalytic site. This mutation, detected in combination with the p.Gln406* change, is linked to the early infantile phenotype with fetal hydrops and cardiac involvement.

*CTSA* gene, spanning about 43.000 bp and containing 15 exons, is about twice the average length of human genes. However, reported CTSA mutations are a very low number. The reason for which a region or site have a higher or lower mutation rate is poorly understood, except in the case of CpG islands where cytosine can become methylated and unstable, leading to a higher rate of mutation [26].

The majority of *CTSA* genetic lesions occur at positions that are evolutionarily conserved [27], and non-variable sites may indicate protein sequences under more selective constraints [28]. Thus, the PPCA/CTSA paradigm could be useful both at genetic level, identifying base composition bias around CpG dinucleotides, distribution and rate of single nucleotide polymorphisms, and at functional level, giving structural consequences of mutated amino acids and regions.

## Conclusions

In early reports mutations nomenclature was selected according to all *CTSA* isoforms (three different isoforms), thus generating a lot of confusion. In order to assist physicians in the interpretation of detected mutations, we underline the correct nomenclature for CTSA mutations.

Four cases with the rare infantile form of galactosialidosis are here detailed and three novel nucleotide changes were identified, one of them resulting in a stop codon, a type of mutation identified for the first time in galactosialidosis.

We also present some data on brain magnetic resonance, never detailed so far in galactosialidosis. Likewise, predictive functional/structural analyses of mutant proteins and phenotypes have been shown to be closely related, often giving clearcut indications on the specific form of the disease (early infantile, late infantile, juvenile).

The complexity of the clinical phenotypes in GS reflects the dual functions of PPCA/CTSA (catalytic and regulating/protective) and thus its functional role in both lysosomal and cell membranes. Further three-dimensional studies can provide additional information on functional domains, on protein-protein interactions within the lysosomal and the non-lysosomal complexes and on the onset and progression of symptoms.

## Abbreviations

CTSA: Cathepsin A; PPCA: Protective protein/cathepsin A; GLB1: Beta-Galactosidase; NEU1: Neuraminidase; GS: Galactosialidosis; LMC: Lysosomal multienzyme complex; GALNS: N-acetylgalactosamine-sulfate sulfatase; LAMP2a: Lysosome-associated membrane protein 2; EBP: Elastic binding protein; MoRFs: Molecular recognition features; BCA: Bicinchoninic acid assay.

## Competing interests

The authors declare that they have no competing interests.

## Authors’ contributions

AC, and AM conceived the study, participated in the genetic studies and drafted the manuscript. SC, and RT participated in the genetic studies, sequence alignments and in the *in silico* analyses. HM and IM have made substantial contribution in the analyses and interpretation of data. MAD, RG and AD participated in the design and coordination of the study. LL and CRP have been involved in revising the manuscript critically. All authors read and approved the final manuscript.
